# Myths of trauma memory: on the oversimplification of effects of attention narrowing under stress

**DOI:** 10.3389/fpsyg.2024.1294730

**Published:** 2024-07-22

**Authors:** Deborah Davis, Alexis A. Hogan, Demi J. Hart

**Affiliations:** Department of Psychology, University of Nevada, Reno, NV, United States

**Keywords:** trauma, memory, sexual assault, memory distortion, tunnel memory, trauma-informed

## Abstract

The present article addresses claims commonly made by prosecution witnesses in sexual assault trials: that attention narrows under stress, and that these attended aspects of the event are encoded in a way that ensures accuracy and resistance to fading and distortion. We provide evidence to contradict such claims. Given that what is encoded is largely the gist of one's interpretation of experience, we discuss the way in which attention and emotion can bias the interpretation of experience. We illustrate with issues of memory reports in cases of acquaintance rape, where the primary issue is the presence or absence of consent. We provide some specific illustrations concerning effects of emotion on interpretation of sexual consent. Finally, based on what is known regarding priming effects on memory retrieval and judgment, we conclude with discussion of the potential of some “trauma-informed” interviewing strategies to promote false memories (such as FETI: Forensic Experiential Trauma Interview).

## 1 Introduction

Many trials of child or adult sexual assault feature two kinds of experts addressing memory: often specifically trauma memory. On the plaintiff or prosecution side will be what is often referred to as a “counterintuitive behavior” expert (Henceforth, CBE). This expert will testify concerning behaviors that seem intuitively inconsistent with having been sexually assaulted, typically suggesting that these are actually common among victims. These include such things as continuing contact with and other seemingly positive reactions toward the perpetrator, long delays in reporting, failure to fight back during the assault, and other behaviors that are subjects of what are claimed to be “rape myths” and other lay misunderstandings of the behavior of victims. Often such experts also testify concerning memory: to explain, for example, why victims' accounts are assertedly often fragmented, incomplete, and inconsistent over time. They also commonly assert that whereas memory for some aspects of a sexual assault might fail or be distorted, memory for the fact of the assault and central details are highly resistant or impervious to failure or distortion. These experts are often clinical or development psychologists, social workers, or law enforcement personnel. On the defense side will typically be an academic expert, often a cognitive or social psychologist, who is called to contest some of the claims of CBEs regarding “rape myths” or “child sex abuse accommodation syndrome” and/or those regarding memory.

The purpose of this paper is to address a specific issue commonly addressed by CBEs: that of the combined effects of attention and emotion on information processing and memory. We focus on the specific claims of many CBEs concerning “tunnel memory,” or attention narrowing under stress, and its effects on memory strength and accuracy. We do not purport to review the full range of issues or research literature regarding trauma and memory. Nor do we attempt to provide full reviews of the specific points we address. We address only the issue of what effects attention exerts on memory in the context of strong emotions. Our goal is to demonstrate that the claims of CBEs regarding combined effects of attention-narrowing and emotion are both oversimplified and inaccurate or unjustifiably extreme.

For example, these claims include the idea that memories formed under stress are accurate regarding the central features of the event: period. Among the experts promoting this claim is Dr. Rebecca Campbell: a psychology professor at Michigan State University. She commonly testifies as a CBE expert, and has offered training to Universities, police departments, and training organizations such as John Reid Associates (one of the premier interview/interrogation training organizations in America) on the “neurobiology of trauma” and its implications for memory of traumatic events such as sexual assault.

Dr. Campbell asserts as follows:

“… memory can be slow and difficult — because the encoding and the consolidation went down in a fragmented way. It went down on little tiny post-it notes and they were put in all different places in the mind. And you have to sort through all of it, and it's not well-organized, because remember I told you to put some of them in folders that had nothing to do with this. But the question everybody wants to know about is the accuracy of that information, okay. And what we know from the research is that the laying down of that memory is *accurate* and the recall of it is *accurate*. So what gets written on the post-it notes — *accurate*. The storage of it is disorganized and fragmented.

So, victims who are assaulted under the influence of alcohol, may not have anything to retrieve. So to speak, their post-it notes are just blank. They may not have it, okay? But for those who are able to remember it, either in pieces or parts, it does go in *accurately*, it does come out *accurately*, but it comes out slow, steady, fragmented, and disorganized….And again, they interpret this victim's behavior as evasiveness or lying. And again, what it really is, most often, is that the victim is having difficulty accessing the memories. Again, the content of the memory the research tells us very clearly is *accurate*. It's just going to take some time and patience for it to come together” (Campbell, 2012).

https://nij.ojp.gov/media/video/24056#transcript$-$0

Similarly, Dr. Jim Hopper is a clinical psychologist who also often testifies as a CBE, guest-writes for popular magazines, and educates Harvard Medical School students about counterintuitive behavior and memory for trauma. He likewise emphasizes the accuracy of memory for traumatic events. He maintains an elaborate website describing his analysis of the neurobiology of trauma and its implications for the behavior and memory of victims (Jim Hopper, Ph.D.).

“Research also shows that usually people *accurately* recall the ‘gist' and ‘central details' of highly stressful experiences” (Hopper, 2023).

It is important to note that nothing in the memory literature suggests that any memories, particularly for complex events, can be presumed accurate. The literature documents factors that increase or decrease the likelihood of accuracy. Moreover, in further contradiction to many such claims, we present evidence that attention does not guarantee accuracy, and instead, particularly in combination with strong emotions, can promote inaccuracy. We include a discussion of how some prominent “trauma informed” strategies of interviewing sexual assault victims can exacerbate memory distortion rather than facilitate accurate retrieval.

As we proceed, we discuss the way in which the potential for memory distortion is important for litigation of claims of disputed sexual encounters between acquaintances, where the primary issue is whether there was or was not consent (or at least a reasonable belief in consent on the part of the accused). In such cases, many details, both historical and surrounding the encounter itself, become important for issues of actual and perceived consent. We suggest that it is unrealistic to assume that remembered details of consent-related communications and other consent-related details that are so important in claims of acquaintance rape will be unerringly accurate for either party. As we shortly review, in no way do emotions, whether rising to the level of trauma or not, guarantee such accuracy: nor do any of the well-established functions of cognition or memory.

Considering the context of acquaintance rape, it is important to address the wide range of emotions that are relevant. Such incidents do not always rise to the level of trauma or intense fear. They can also be an issue of annoyance, provoking emotions such as irritation, anger, embarrassment, or disgust. Moreover, the accused's emotions are relevant as well: and can include those such as sexual arousal, or happiness. As we consider in sections to come, different emotions can have different effects on processing and interpretation: and thus emotion-related arousal will function somewhat differently in different emotional contexts.

It is also important to emphasize that regardless of what emotions an accuser might experience during a sexual encounter, no matter how extreme the emotions are, and no matter how firmly she is convinced that she was raped, the event may not qualify legally as rape. This judgment will rest in part on the nature of the interaction itself and features of the context going to the issue of what a reasonable man in the situation would believe regarding consent. The woman can absolutely feel that she was raped, even though her consent-related communications and behaviors could have allowed a reasonable belief that she consented (see Wood et al., 2019 for a review of legal standards of consent). As some studies have indicated, there can be a discrepancy between internal feelings of consent and external expressions of it (e.g., Willis et al., 2019). Likewise, a man can have an unjustified view of consent, believing there was no rape even though legal standards would suggest there was. Given the wide range of consent-related details relevant to claims of acquaintance rape, it is crucial to understand how emotion can cause predictable forms of distortion for their memory.

We begin our discussion with a brief review of the encoding function of memory and the determinants of what is encoded. Next, we turn to a brief review of the concept of tunnel memory and claims regarding the effects of attention narrowing under stress. We then explore the way in which claims regarding emotion, tunnel memory, and accuracy are often oversimplified and misleading. We specifically address (1) difficulties in the prediction of what attention will narrow to, (2) how attention affects information processing, (3) how these processes are exacerbated and/or modified by emotion, (4) why predictable failures and distortions in memory encoding can result from attention itself and its combination with emotion, and (5) the importance of consideration of post encoding processes promoting distortion. We consider these issues in the context of claims of counterintuitive behavior experts regarding memory for alleged sexual assault. As context for these discussions it is important to emphasize that much of the social interaction that is relevant for judgment of disputed sexual interactions is not necessarily stressful for either participant. Particularly in cases of alleged date rape, the interactions that convey likely or actual sexual consent can take place across substantial time in advance of the specific actions alleged to be assault. Accordingly, the claims of CBEs regarding memory for trauma are irrelevant to much of the information sought from the parties concerning potentially consent-related behaviors and communications leading up to the disputed actions: though the many effects of specific emotions on cognition documented by cognitive scientists can be very relevant.

## 2 What is encoded?

Errors in memory are not just about the process of “remembering.” They begin with the initial processes of perception during the event and how event gist and details are encoded into memory. Though this point is second nature to cognitive psychologists, as context for discussion of why accuracy in encoding is never guaranteed, it is nevertheless worth emphasizing that what is initially encoded includes some verbatim representations, along with the gist of our interpretation of what is attended to about the event. Over time, verbatim images progressively fade and memory favors the gist of our interpretation of what was attended to during the event.

In contrast to the myth endorsed by many among the public, and even by some professionals, our observations and memory do not work like a video camera. Our observations of any given situation are selective, in that we do not attend to all aspects of an event. Moreover, memory follows the focus of attention, such that the more something is attended to, and the greater the processing devoted to it, the more likely it will be remembered. Finally, and most importantly for our discussion to come, encoding is “interpretative:” in that an interpretation is imposed on what is observed, and that interpretation is encoded along with the gist of the surface characteristics of what is interpreted. That interpretation might be simple categorization: such as “bear,” “table,” “man,” or “soldier.” Or it might be a characterization of behavior (such as “hostile,” “coercive,” “consenting,” or “resistant”) or emotion (such as “happy,” “sad,” “afraid,” or “embarrassed”: for reviews of these principles see Davis and Loftus, 2016; Reisberg and Heuer, 2020; Davis et al., 2023). It is these functions of selectivity and interpretation that pose the potential for inaccuracy and so soundly contradict the idea that memory accuracy can be guaranteed (or almost guaranteed) under any circumstances. As we discuss in sections to come, the interplay of emotion with processes of selective processing and interpretation provides a double-edged sword: in some ways enhancing the strength of memory for an experience, but also risking greater error in what interpretation of that experience is recalled.

## 3 Fundamentals of the tunnel memory hypothesis

The fundamentals of the tunnel memory hypothesis (originally called the “Easterbrook Hypothesis”) were first proposed by Easterbrook (1959). Easterbrook suggested that the arousal associated with emotion causes a narrowing of attention to “central” aspects of an event (and therefore better memory for central information), at the expense of attention to “peripheral” aspects (and therefore poorer memory for peripheral details). The phenomenon was later dubbed “tunnel memory,” and the phrase is now frequently employed to depict this severe and concentrated focusing of attention on specific facets of a situation (Mackworth, 1965; Safer et al., 1998).

There has been some theoretical debate concerning the specific cause of emotion-related attention narrowing. Whereas, Easterbrook (1959) viewed the arousal associated with emotion as the cause, others have pointed to such possibilities as defensive strategies that direct attention (such as the disassociation that has been suggested to occur in rape victims (Brokke et al., 2022; Lynch et al., 2023); or the fact that “attention magnets,” or stimuli that naturally draw attention (such as horrific violence) can both cause attention to narrow to themselves and also cause the emotion, rather than the reverse cause where emotion causes narrowing of attention to the stimulus (see Reisberg and Heuer, 2020 for review). Nevertheless, there is considerable empirical support for the proposition that emotion is associated with the narrowing of attention (Levine and Edelstein, 2009; Mitchell, 2023), and general agreement between memory scientists and both defense and prosecution experts on this point. The disagreement concerns the direction and consequences of that narrowing.

## 4 Why stop there? Oversimplification of effects of attention and emotion

The simple view of emotion and attention narrowing suggests that information that is attended to will be more successfully encoded into memory. Clear support exists for the idea that the gist of emotional events is more successfully encoded into memory, and there is general agreement concerning the resistance of memories for highly emotional events to forgetting. Indeed, Daniel Schacter has included this resistance, dubbed “persistence,” as one of his “seven sins of memory” (Schacter, 1999, 2001; see also Bonsall and Holmes, 2023): though it might be considered a “sin” mostly for negative or traumatic events that are resistant to efforts to forget.

However, as previously established, counterintuitive behavior experts, such as Dr. Rebecca Campbell, Dr. Jim Hopper, and others, further argue that this information will be encoded accurately, and that it will be highly resistant, if not completely impervious, to both fading and memory distortion (Hopper, 2018a).

For example, Dr. Bessel van der Kolk, psychiatrist, author, and frequent CBE expert asserts:

“What is so extraordinary about trauma is that these images or sounds or physical sensations *don't change over time*. So people who have been molested as kids continue to see the wallpaper of the room in which they were molested. Or when they examine all these priest-abuse victims, they keep seeing the silhouette of the priest standing in the door of the bathroom and stuff like that. And so it's these images, these sounds that don't get changed” (Tippett, 2021).

https://onbeing.org/programs/bessel-van-der-kolk-how-trauma-lodges-in-the-body-revisited/

Such claims as these suggest that the accuracy of the accounts of alleged victims should not be questioned, but rather should be presumed almost certainly accurate. We suggest, in contrast to this simple view, that the combined effects of emotion and attention are more complicated. In the sections to come we consider a number of processes that can instead undermine the accuracy of information encoded in the context of specific emotions.

### 4.1 What is actually encoded?

Given that attention provides the opportunity for encoding, what is it that actually gets encoded? Memory scientists generally agree that this consists of the gist of one's interpretation of what was attended to (e.g., Reisberg and Heuer, 2020). Whereas, verbatim images can be included during encoding, they are nevertheless interpreted, and it is the gist representations that persist more strongly over time. Therefore, it is important to ask what determines what is attended to and how it is interpreted. As we discuss in the sections to come, what is encoded is not necessarily what is legally most relevant, as many CBEs state or imply. Nor is the encoded interpretation always accurate.

### 4.2 Where does attention go under stress?

A necessity for predicting which features of an emotional event will be remembered is accurate understanding of where attention will go in the circumstance. This point has been central to understanding why, for example, the presence of a weapon has led to poorer memory for the face of a criminal perpetrator. Attention goes to the weapon instead, and therefore the face is remembered more poorly (Loftus et al., 1987; Steblay, 1992; Pickel, 1999).

Research has indicated that it is more difficult to identify what might be “central” than many contemplate. For example, emotions experienced during a threatening event are assumed to direct attention toward the aspects of the event that we determine to be the most useful for survival in the moment. More generally, Levine and Edelstein (2009) and Kaplan et al. (2012) have noted that specific emotions tend to activate specific goals, and to direct attention toward goal-relevant information (see also Fredrickson, 2000; Levine and Pizarro, 2004; Huntsinger, 2012, 2013; Harmon-Jones et al., 2013). The valence of emotions can also direct attention to emotion-consistent or inconsistent stimuli, depending upon the emotion-provoked goals in the situation (e.g., Clore et al., 2018; Clore and Schnall, 2019; Yu et al., 2021).

In some cases, emotion can direct attention away from, rather than toward, stimuli: such as when disgust directs attention away from the disgust-provoking stimulus; when shame directs attention away from a rapist's face; or when attention is directed away from the sexual activity itself in order to suppress extreme emotions (as is claimed regarding the tendencies of rape victims to disassociate: Kindelan, 2018). Emotion regulation strategies provoked by extreme emotions can have strategy-specific directive effects on attention and interpretation (such as distancing vs. reappraisal: e.g., Schmidt et al., 2010). In others, the goal might be mood maintenance, and therefore selective attention toward mood-consistent, and away from mood-inconsistent, information. Where details of an interaction become crucial to disputed sexual events, it is important to note that attempts to suppress the outward expression of emotions have been shown to impair memory for factual details of an interaction but increase memory for emotional reactions: presumably, because attention is directed toward the emotions and away from the interaction (Richards and Gross, 1999, 2000, 2006; Chang et al., 2018). Such findings suggest that it is no simple matter to identify what aspects of a disputed sexual encounter would have been attended to.

Again, it is important to note that “trauma” is not likely to occur during all time periods relevant to an alleged victim's account. For example, whereas details concerning interactions between accuser and accused leading up to the sexual encounter can include many consent-relevant communications and behaviors, the trauma itself (if any) should begin during that encounter or when it becomes clear that unwanted sex will occur. The timing of the trauma relative to the to-be-remembered information is crucial, in that any of the effects of the purported “neurobiology of trauma” on memory should not include pre-trauma information (see Marr et al., 2021 regarding effects of timing of stress relative to to-be-remembered information). But what has research shown us about where attention goes during a sexual assault? Anecdotal reports point to a fairly large variety of targets of attention during alleged sexual assaults. For example, sexual assault survivors often recount instances of tunnel memory, wherein they might recall only specific details such as the expressions on the perpetrator's face, the smell of his cologne, or the sound of his voice, the wallpaper in the room, upholstery in the car, the weapon they were carrying, sensory perceptions such as sound or smell, and many more details that might seem peripheral: rather than, or, in addition to, the actions of the perpetrator and the rape itself (Steblay, 1992; Percy, 2023).

A common viewpoint expressed by many CBEs is that rape victims tend to disassociate during the event, paying attention to anything but the assault and resulting feelings (van der Kolk, 2014; Hopper, 2015; Kindelan, 2018).

According to Dr. van der Kolk: “Dissociation is a temporary putting aside, not knowing, and not noticing. It's a way to survive. Blocking things out allows many traumatized people to go on. It may be very helpful in order to make it through the crisis, but in the long-range, living your life in a dissociative way only keeps the trauma alive” (Melaragno, 2018) (https://www.dailygood.org/story/1901/trauma-in-the-body-an-interview-with-dr-bessel-van-der-kolk-elissa-melaragno/; https://www.besselvanderkolk.com/resources/the-body-keeps-the-score).

Assuming, however, that disassociation does not occur for a particular accuser, where might attention go? Each party's behaviors might be neglected by an accuser if disassociation occurred. But either way, where might each party's attention be focused? While there might be commonalities in the focus of attention between people, there are also likely individual differences. Some might focus on their own emotions and sensations. Others might devote more attention to the behaviors and reactions of the other person. Still, others might experience wide-ranging divided or rapidly shifting focus of attention (e.g., Kern et al., 2005). Attention might be focused on the behaviors of the other person leading up to sexual activity, in an effort to read the other's interest and intentions: and become more self-focused when sexual activity commences. If the woman feels that the encounter is unwanted, she may focus on how to escape. The possibilities for allocation of attention are extensive, making generalizations concerning what behaviors will be “central” and most likely remembered as inappropriate. Arguably, the specific behaviors going to a reasonable belief in consent may be less likely remembered than the feelings generated (recall the common advice that while you might not remember everything a person says or does, you will remember how they made you feel).

The issue of what is remembered is, of course, central to litigation of disputed claims of sexual assault. It is unfortunate that many of the most crucial details for litigation might not have drawn attention during the event or later be remembered. This issue is particularly important with respect to the example of alleged acquaintance rape, litigation of which arguably demands consideration of more wide-ranging information than that of stranger rape.

For the sake of any subsequent reports and associated litigation, a crucial set of issues for allegations of acquaintance rape concerns the behaviors of each party going to whether consent did or did not occur. Were there coercive behaviors? What behaviors occurred leading up to and during the event that indicated consent vs. refusal?. Were there alcohol or drugs involved? If so, when and how much did each person ingest? As previously noted, a person can feel that she was raped, even if the situation did not meet the legal standards for the crime: for example, if the accused could have a “reasonable” belief that the woman had consented (People v. Mayberry, 1975). To understand this, it is important to have accurate information concerning what was said and done by each party leading up to and during the disputed encounter.

Clearly, many details that might be considered important for litigation would not be the primary “attention magnets” during the period leading up to and during the event. These details may be encoded only vaguely or not at all: leaving them more susceptible to distortion based on context or suggestion. As shown by research on “fuzzy trace theory,” contextual and suggestive influences on memory exert greater impact when the original encoding is more vague, or when the original traces become more vague over time (e.g., Brainerd and Reyna, 2019; Bialer et al., 2021; Brainerd et al., 2021, 2022).

Before leaving this discussion of attention, we note that it is important to consider, as well, that while both academic and legal attention is most often devoted to the emotions and memory of the accuser, those of the accused are important for understanding what he will remember and report (and what might be reasonable beliefs regarding consent for a person in his shoes).

### 4.3 What does attention do?

At the most basic level, attention provides the opportunity for encoding. This opportunity, however, does not guarantee retention. Information encoded into short term memory does not necessarily reach long term memory: as can occur with alcohol blackout (Lee et al., 2009), with head trauma (Vanderploeg et al., 2014), with sufficiently high levels of stress (Trammell and Clore, 2014), or with superficial attention (Schacter, 1999, 2001).

Retention is most likely to occur with elaborative encoding: where the person thinks about what is observed, forming more links to other information in memory that can later facilitate retrieval (Coane, 2013). Indeed, even early academic discussions of tunnel memory noted the relationship of narrowed attention to elaborative processing:

“Participants comprehend a neutral scene by automatically extending its boundaries and understanding the visual information in a broader external context. However, when participants are negatively aroused by a scene, they process more elaborately those critical details that were the source of the emotional arousal, and they maintain or restrict the scene's boundaries. ‘Tunnel memory' results from this greater elaboration of critical details and more focused boundaries. Tunnel memory may explain the superior recognition and recall of central, emotion-arousing details in a traumatic event, as shown in previous research on emotion and memory” (Safer et al., 1998, p. 116).

However, elaborative encoding does not ensure a reliably objective representation of what occurred. When attention is brought to bear on something, the observer's general knowledge and expectations affect what is subjectively perceived. As perception theorists have routinely demonstrated, perception is inherently constructive, adding to what is physically perceived, filling in with what is expected (e.g., Hoffman, 2019). Cognitive psychology has further demonstrated the effects of expectations on perception of physical objects: showing, for example, that the same physical object can be seen as a rabbit vs. a duck, a number vs. a letter, or an old witch vs. a young girl depending upon which concept or expectation is activated. They have likewise argued that without such expectations we would not even know how to label what we observe or how to react to it (Brosch et al., 2013; Hoffman, 2019). Moreover, illustrations within all areas of psychology abound of “cognitive bias” in interpretation due to chronic and situationally activated expectations (Lord and Taylor, 2009).

In these respects attention can be regarded as a double-edged sword, in many cases affording us the opportunity to accurately understand and encode what we experience: but at the same time serving as a potentially biasing machine, leading us to slant the interpretation of what we experience toward consistency with salient expectations.

### 4.4 What does emotion do?

Beyond the attention narrowing effects of emotion, counterintuitive behavior experts often testify to a number of additional effects of stress on encoding. These include, for example, claims regarding the physiology and “neurobiology” of responses of victim-survivors to trauma: such as their effects on memory. These claims tend to be a mixture of fact and fiction. Many claims regarding how emotion-provoked physiological/neurological responses potentiate encoding are empirically supported, though a review of these is beyond the scope of this review. In essence, though, research has shown that emotion and “trauma” tend to amplify the strength (as distinct from the accuracy) of encoding of the person's interpretation of the gist of the attended aspects of the experience (Brosch et al., 2013; Schoch et al., 2017), and this basic effect of strong emotions is relatively uncontested^1^.

However, CBEs tend to go beyond the potentiation of encoding to claim that what is encoded is accurate, and so strongly encoded as to defy subsequent distortion: Hopper (n.d.-b) as reflected in the quotes of CBEs Rebecca Campbell and Jim Hopper provided earlier (Campbell, 2014; Hopper, 2023).

Such claims belie the basic truth that what is encoded is the gist of the interpretation of the attended information. Moreover, they ignore research concerning the impact of emotion on interpretation, and the implications of this for accuracy. Generally, work on implicit associations has shown that mental associations activated, even outside awareness, shape judgments outside of awareness (e.g., Greenwald and Banaji, 2017). In part, context affects which associations are activated, and thereby inevitably affects interpretation. Emotion is one feature of context, and as such also affects interpretation: in part through associations between emotion and the conditions that tend to produce them (such as when a person interprets an event in a way that explains his or her emotional reactions: Davis et al., 2023). Research has pointed to at least three mechanisms through which emotion affects interpretation.

#### 4.4.1 Affect as information

First, through the “affect as information” mechanism, the person may interpret the event or aspects of the event in a way that makes sense in light of current emotions. The person's own emotional reaction is the primary basis of judgment, without reliance on all potentially relevant additional information in the situation. This mechanism of influence is more likely to occur in circumstances that likely apply in most disputed sexual encounters, as well as in uncontested instances of rape: when emotions are strong, when emotions are produced in the situation that is to be judged, when there is room for interpretation, and when intense processing is applied to the judgment [see reviews by Greifeneder et al. (2011), Clore et al. (2018), and Clore and Schnall (2019)].

#### 4.4.2 Affective priming

The second mechanism is “affect as context” or “affective priming.” This mechanism was identified in the Affect Infusion Model of Forgas (2002). In this view, emotion serves to contextually activate emotion-consistent schemas and expectations that direct the processing of information and affect judgments. Consistent with the AIM model, emotions exert more impact on judgments when the person engages in more elaborative processing of the event: such as one might expect of sexual assault victims (at least after the fact, if not during). As Davis et al. (2023) pointed out, emotions that can be provoked in an unpleasant sexual encounter might include those such as irritation, resentment, anger, fear, and disgust, which can be associated in memory with concepts of coercion or rape: thereby potentially biasing interpretation and memory toward consistency with rape.

As with any form of priming, emotions can also prompt constructive memory processes such that individuals can supplement their recollections with what they anticipate ought to exist in the surrounding context, guided by their emotion-related schemas and scripts. As Brainerd et al. (2008) have noted, the activation of a network of emotional connections can lead to intensified conceptual priming and an elevated feeling of familiarity, promoting both accurate and erroneous memories of emotional stimuli.

#### 4.4.3 Emotion and intuitive processing

A third effect of emotion concerns the disengagement of System 2 (More Elaborative, Analytical, Reflective) processing (Davis and Loftus, 2009; Kahneman, 2011). To put this more strongly, emotion can impair frontal lobe functioning: generally leading the person to rely more strongly on ingrained habits and instinctual behaviors. Similarly, some CBEs and defense experts agree that habitual behaviors, such as behaviors informed by instinct, schemas, and scripts, can be rendered more dominant during a traumatic experience due to the impact of stress on the brain regions responsible for regulating our thoughts, emotions, and actions.

For example, Dr. Jim Hopper asserts: “When the fear circuitry kicks in [during the midst of a sexual assault], basically there's a subnucleus in the amygdala (a brain region responsible for emotional processing and connecting emotions to memory) called the central nucleus and it sends a signal to the brain stem that says ‘hit the prefrontal cortex with more norepinephrine and dopamine.' So the fear circuitry triggers chemicals that hit the prefrontal cortex (a brain region responsible for regulating rational thoughts, actions and behaviors) and impair it.”

(Hopper: Sexual Assault & the Brain in 6 minutes, 2018; Hopper and Lisak, 2014).

As Dr. Hopper further explains, “When a larger predator is coming at you or has you in its grip, thinking through a response with your rational prefrontal cortex is too slow and could get you killed. But reflexes and habits, which your brain can automatically cue up and execute in fractions of a second, could save your life. So evolution selected brains in which stress and trauma impair the prefrontal cortex, because that allows fast reflexes and habits to take over.”

(Hopper: Sexual Assault & the Brain in 6 minutes, 2018; Hopper and Lisak, 2014).

CBE Lisak also offers an elaborate discussion of the stress-induced disengagement of the frontal lobes and the implications for habitual behaviors (Neurobiology of Trauma—Dr. David Lisak—YouTube) (Lisak, 2013).

Interestingly, however, Dr. Hopper, Dr. Lisak and other CBEs stop short of recognizing the effects of the dominance of habits on cognitive functions. Habits of the mind are equally promoted by the disabling of executive functioning: shunting the person into System 1 modes of intuitive thought driven by habits, schemas, and expectations (Davis and Loftus, 2009). According to Kahneman (2011), System 1 operates automatically and quickly without conscious effort or voluntary control. In this mode, a person's initial impressions of what they are experiencing go uncorrected by the more deliberate, rational analysis carried out in System 2 (Kahneman, 2011). This is because the automaticity of System 1 cannot be turned off at will, so any perceptual errors that occur during this stage are difficult to prevent. The correction of errors is left up to the slow, enhanced monitoring of System 2. However, many errors go unnoticed and uncorrected, because it's impractical to constantly question the accuracy of one's own thinking. Thus, we place trust in the snap decisions made by System 1 (Kahneman, 2011). Since emotion serves as the context triggering the expectations and associations served up by the intuitive system and used for interpretation, these interpretations are likely to be at least somewhat biased by the emotions.

## 5 What do we know about the role of emotion in interpretation and judgment of sexual consent?

A large literature has accumulated regarding how sexual consent is conceptualized, conveyed, and interpreted (see Wertheimer, 2003; Muehlenhard et al., 2016; Fenner, 2017; Wood et al., 2019; Kabota and Nakazawa, 2022 for reviews). This literature includes an array of studies designed to understand sources of miscommunication of consent. Among these are cultural scripts that promote misunderstanding: such as belief in “token resistance,” whereby women may say “no” when really meaning “yes”: offering “token” refusals before consenting to sex (e.g., Muehlenhard and Hollabaugh, 1988). Also included are studies of individual differences in beliefs that underlie many misunderstandings: such as traditional sex role beliefs, rape myth acceptance or “rape supportive attitudes”, and others: many of which are also predictive of verdicts in trials of sex crimes (see Ryan, 2011; Rerick et al., 2019 for reviews). These comprise an array of cultural, perpetrator, victim and situational variables underlying miscommunication of consent and/or perpetration or victimization (Adams-Curtis and Forbes, 2004).

While many studies have addressed how and why women can fail to communicate resistance effectively, studies of errors in the interpretation of consent behaviors have been almost entirely restricted to males. There is, of course, considerable concern with why men coerce women, and it is assumed that the misperception of consent cues is among the causes. Accordingly, studies have addressed issues such as whether men on average tend to “overperceive” consent relative to the actual intentions of women; which female behaviors are most commonly perceived as indicating consent vs. refusal; which men are most prone to overperceive cues of consent; and what circumstances promote such misperceptions (see Wertheimer, 2003; Muehlenhard et al., 2016; Fenner, 2017; Wood et al., 2019; Kabota and Nakazawa, 2022 for reviews). These sorts of studies are crucial to understanding sources of disagreements between accusers and accused concerning whether consent did or did not occur. However, they also illustrate how vulnerable sexual interactions are to subjective interpretation, and how precarious are assumptions that reports of these interactions will be fully accurate on either side.

Nevertheless, much remains to be addressed. Studies of misperceptions of sexual consent have almost exclusively focused on the misperceptions among males of female consent. We are unable to locate studies focusing on female misinterpretation of coercion or of accuracy in understanding the clarity of their own sexual consent behaviors and communications. Memory reports of these behaviors by accusers are crucial to judgments of claims of sexual assault, and as such, research is needed to address the personal, situational, and cultural forces that might compromise accuracy.

Additionally, almost no research has addressed the manner in which emotions can impact the interpretation and memory of sexual consent interactions. Given that the issue of whether there was or was not consent to sexual activity is central to disputes regarding acquaintance rape, it is important to know how emotions might specifically affect judgments and memory of sexual consent. To date, little research has investigated this topic, particularly as it concerns female interpretation/memory of coercive male behaviors or their own behaviors communicating consent or non-consent.

## 6 Research on emotion and judgment of sexual intentions

Davis et al. have conducted a series of studies relevant to the effects of emotion on judgments of female sexual willingness. Though we have recently begun to study perceptions of male potentially coercive behaviors, these largely concern emotions likely experienced by the initiator of sex: and perhaps only the accused when there is a disputed sexual assault.

### 6.1 Sexual arousal

Several of these concerned the impact of male sexual arousal on interpretation of the extent to which specific female behaviors implied sexual willingness. The authors suggested that sexual arousal might lead aroused men to infer that specific behaviors reflect greater sexual interest or willingness compared to unaroused men (see also Murray et al., 2017). Several lines of research support such a prediction.

Steele and Josephs's (1990) theory of “alcohol myopia” proposed that alcohol narrows attention to impulse consistent cues and inhibits attention to impulse inconsistent inhibitory cues. Similarly, in line with the previously cited work on attention to emotion related goals, Loewenstein (1996) argued that strong emotions can lead the person to focus on how to resolve or satisfy the emotion quickly, without full consideration of reasons to avoid the behavior in question; or to focus attention inwardly and compromise concern for others. Similar in its effects to “alcohol myopia” (Steele and Josephs, 1990), sexual arousal could lead the person to rely on promotional cues (favoring sexual activity) more strongly than inhibitory cues (disfavoring it). To the extent that sexual arousal narrows attention to cues consistent with sexual activity, or biases interpretation of all cues in that direction, one would expect arousal to promote stronger perception of sexual consent.

Across three studies, Davis and colleagues asked male participants to either write an arousing sexual (vs. non-sexual) fantasy, or to view relatively arousing (vs. non-arousing) pictures of females. They were then asked to rate the extent to which each of 25 specific behaviors reflected sexual willingness (though the specific questions regarding willingness varied). In all studies, sexually aroused males (particularly single males) rated the female behaviors as reflecting greater sexual willingness (Livingston and Davis, 2020; Rerick et al., 2020). Relatedly, Bouffard and Miller (2014) found that though manipulated sexual arousal did not affect ratings of female sexual willingness in a dating scenario, self-reported sexual arousal did do so. Perhaps likewise reflecting sexual motivation, Rerick and Livingston (2022) found that specific behaviors were perceived as reflecting greater sexual willingness for attractive than unattractive women: and this effect was mediated by sexual arousal.

Miller and Davis (2024) recently found that arousal among both men and women was associated with perceptions that specific female behaviors reflected sexual willingness: as well as that they did so more strongly if toward a man who was attractive and had stronger financial credentials. However, arousal itself (while reading about and reacting to the female behaviors) was strongly predicted by political conservatism and religiosity. As these characteristics are associated with many of the rape-supportive attitudes mentioned earlier (such as rape myth acceptance, belief in token resistance to sex, and others e.g., Bohner et al., 2009; Rerick et al., 2019) it may be that effects of individual differences in the tendency to get sexually aroused when reading about sex-related behaviors (and therefore self-reported arousal) are actually reflecting effects of individual differences in rape supportive attitudes. However, this relationship might well be bi-directional, in that Rerick et al. (2022) found that sexual arousal led to greater agreement with attitudes consistent with greater permissiveness for sexual activity: including those regarding female token resistance to sexual activity, and assertive sexual strategies.

Indirect evidence is also consistent with such biasing effects of sexual arousal. Sexual arousal has been shown to shift motivation away from longer term desires toward satisfaction of more immediate ones (see Kim and Zauberman, 2013 for review). Sexually aroused males find females more attractive (e.g., Stephan et al., 1971; Ditto et al., 2006), find female faces to reflect greater sexual arousal (e.g., Maner et al., 2005), and find sexual material less disgusting (e.g., Stevenson et al., 2011). They also report greater willingness to engage in forms of sex they might otherwise find unacceptable: such as sex with unattractive or older women, sex without protection, or inappropriate coercive behaviors (Blanton and Gerrard, 1997; Ariely and Loewenstein, 2006). Such findings are consistent with the notion that sexual arousal facilitates perception of the social world as consistent with sexual activity: which would include perceiving potential partners as willing. And, not surprisingly, sexual arousal is associated with sexual disinhibition (e.g., Bouffard and Miller, 2014; Imhoff and Schmidt, 2014).

Our lab has recently begun to study determinants of perceptions of the coerciveness of male behaviors. Hogan et al. (2024) asked male and female participants to rate the extent to which a set of potential male behaviors seeking a date or sex put pressure on the female and their appropriateness in the circumstances depicted. Mirroring the Miller and Davis findings, the authors found that among both males and females male behaviors were seen as exerting less pressure and as more appropriate if the male was depicted as physically attractive and as possessing better financial potential. Self-reported sexual arousal predicted these ratings as well. Additionally, political conservatism, religiosity, and endorsement of rape-supportive attitudes predicted both arousal while reading about the behaviors and ratings of pressure and appropriateness. Such strong situational (characteristics of the male involved) and individual attitudinal differences in interpretation of sexual consent -related behaviors undermine the idea that memory for sexual assault incidents will be inevitably accurate.

### 6.2 Effects of felt power

While sexual arousal is perhaps the most pervasively present emotion in sexual encounters, other feelings can also affect interpretation of sexual willingness: among them, “felt power.” Felt power is defined as a person's sense of agency and ability to exert influence over others (Fiske, 1993; Galinsky et al., 2003; Guinote, 2007, 2010; Guinote and Vescio, 2010).

Research has indicated that felt power exerts a number of effects that can become relevant in sexual interactions. Among the most relevant of these are the tendencies to selectively attend to and notice cues consistent with goal pursuit, to fail to notice or ignore goal-inconsistent cues, to interpret social situations as consistent with one's goals, and to feel more confident. Generally, these tendencies, and the power-induced disinhibition they entail, lead persons to more likely and vigorously pursue their goals (Galinsky et al., 2003, 2008, 2016; Keltner et al., 2003; Guinote, 2007, 2010; Lammers et al., 2008; Smith and Bargh, 2008; Guinote and Vescio, 2010; Slabu and Guinote, 2010; Hirsh et al., 2011; Whitson et al., 2013; Pike and Galinsky, 2020).

Though power-induced disinhibition can lead to prosocial behaviors when those are the preferred goals, it has also been shown to lead people to cheat, steal, or violate traffic laws, and generally to disregard social norms (see Lammers et al., 2015 for review): perhaps partly the result of increased tendencies toward social distance from others (see Magee and Smith, 2013 for review). A number of these norm-violating behaviors include sexual behaviors. For example, power increases infidelity among both men and women (e.g., Lammers et al., 2011). Moreover, power is associated with positive reactions (e.g., sexual arousal) to counter-normative sexual behaviors, such as sadistic behaviors among women and masochistic behaviors among men (Lammers and Imhoff, 2016): and is associated with perceptions and expectations of sexual interest from subordinates (Kunstman and Maner, 2011), sexual harassment-consistent cognitions (Pryor and Stoller, 1994; Bargh and Raymond, 1995; Bargh et al., 1995), and sexual aggression (Zurbriggen, 2000).

Based on findings regarding the effects of felt power on selective attention to goal-consistent cues, and biased interpretation of those cues toward consistency with goal pursuit, one might predict that in sexual situations this would include bias toward cues indicating that a potential sexual partner is willing. Consistent with this expectation, Livingston and Davis (2020) showed that males, but not females, regarded specific female behaviors as more indicative of sexual willingness when they were primed to feel more powerful.

### 6.3 Effects of alcohol

The relationship of alcohol to sexual behavior has been extensively studied. In part, alcohol can promote sexual arousal, sexual motivation, sexual activity, and sexual pleasure (see Davis and Loftus, 2004): but it has also been heavily implicated in sexual coercion and victimization (Villalobos et al., 2016; Caamano-Isorna et al., 2021; Steele et al., 2022). Of greatest interest for the purposes of this review are its effects on attention and interpretation in sexual situations.

In this respect, alcohol shares much with both felt power and sexual arousal. Each fuels alterations in cognition and promotes disinhibition. Moreover, they do so through largely comparable mechanisms: as elaborated by Steele and Josephs (1990) in their theory of “alcohol myopia” (see also Davis and Loftus, 2016), by Hirsh et al.'s (2011) analysis of effects of alcohol and power, and by Imhoff and Schmidt (2014) in their application to sexual arousal.

Each affects attention, narrowing it to cues consistent with motivations that are salient in the situation, reducing the depth of processing of the cues that are attended to, and reducing the ability to access existing knowledge and relate it to incoming information. They thereby reduce the complexity with which an event is processed and the extent to which all relevant information is brought to bear on a particular judgment or decision.

Analyses of the effects of power, strong emotion (including sexual arousal), and alcohol all point to the importance of reduced functionality or use of executive functions associated with each (Steele and Josephs, 1990; Hirsh et al., 2011; Imhoff and Schmidt, 2014; Davis and Loftus, 2016). As Daniel Kahneman put it, when executive functions are impaired, such as occurs with alcohol and strong emotions, judgment is based on the assumption “WYSIATI” (“What you see is all there is!” Kahneman, 2011). As such, mistakes in interpretation become more likely, and biased in a direction consistent with the emotion and motivation in question.

In addition to these processes, Davis and Loftus (2004, 2016) noted the relevance of “alcohol expectancy” effects regarding the tendency for alcohol to promote sexual motivation and activity (see also Villalobos et al., 2016; Wood et al., 2019 for review). Because alcohol is expected to promote interest in sex, many report using alcohol as a tool of seduction. Both sexes perceive intoxicated others as more sexually aroused, easy to seduce, and willing to consent. Women report being more likely to use alcohol when willing to have sex, and men believe the same. Reflecting such assumptions, those accused of sexually assaulting intoxicated alleged victims are less likely to be convicted (see Rerick et al., 2019 for review). These expectancy effects would increase the degree to which sexual motivation is assumed among intoxicated others. Moreover, when executive functions are impaired, as they are with alcohol and strong emotions, expectancies exert more influence on judgment (see Kahneman, 2011 for review).

As Davis and Loftus (2016) noted, during a sexual encounter these processes of selective attention and impaired executive functions can lead the perceiver to ignore contextual cues that should inform interpretation of both persons' behaviors and the degree of consent vs. coercion involved: such as the other's level of intoxication, the relationship history between them, the context of their current encounter, behaviors immediately preceding initiation of sexual activity, historical information about each person's behaviors and preferences while not intoxicated, one's own behaviors and effects of those on the other person and much more. Conflicting reports concerning disputed sexual consent can be based in these processes of selective attention, incomplete consideration of relevant information, and biased interpretation reflecting goals of sexual engagement vs. avoidance of the encounter.

## 7 But what about negative emotions associated with trauma?

At present we are not aware of studies examining the influence of negative emotions on judgments of sexual consent. Nevertheless, there is reason to expect that emotions experienced during a sexual encounter or later when recalling it will affect judgments of consent. Davis et al. (2023) have recently outlined reasons for such an expectation, as well as some pathways through which negative emotions can lead to false memories and false allegations of sexual assault.

The authors first suggested that several negative emotions can occur, even during subjectively or legally consensual sexual encounters. Surely, they will occur in instances of genuine rape. However, the authors argued that there are two other common circumstances that can generate negative emotions, such as anger, fear, or disgust: (1) when the person voluntarily chooses to engage in sex when they would prefer not to (such as to please a partner, to avoid conflict, pity sex with an unattractive partner, to secure other benefits from the partner, and others); and (2) when the person does not want to have sex and does not subjectively consent, but does not effectively communicate refusal to the other person in a way a “reasonable person” would understand.

The authors further suggested that negative emotions felt during such encounters can color the interpretation of the interaction such that it is interpreted in a way that makes sense, given the emotions felt. One such interpretation that might explain such emotions could be that the encounter was actually involuntary and/or that the actions of the partner were coercive. Such effects would be consistent with the known mechanisms through which emotions affect judgment discussed earlier. Also as discussed earlier, such emotion priming can affect memory and judgment at the time of the encounter, or later when it is recalled.

Unfortunately, at this time we were unable to locate studies directly addressing the role of negative emotions in judgments of either female consent or male coercion. This is a gap in the literature in need of research. Our lab is in the process of initial efforts to test such effects. Meanwhile, there is some research consistent with the hypothesis that negative emotions such as fear, anger and disgust are likely to affect judgments of consent vs. coercion.

Some such research has come from the domain of moral psychology (e.g., Haidt, 2012). Emotions can be provoked by moral judgments, they can intensify moral judgments, or they can provoke moral judgments for morality neutral behaviors (Haidt, 2001, 2012; Avramova and Inbar, 2013; McAuliffe, 2019). Each of these effects can occur in sexual encounters. Strong emotions can be triggered by the encounter, and the encounter can produce moral judgments for each person's behavior. Such judgments can be made for behaviors that would generally be considered moral violations (such as sexual coercion, infidelity, incest, and others), or be imposed on behaviors that might be considered morally neutral in the absence of the emotion (many objectively non-coercive or permissible sexual invitations, advances, or activities between adults). A number of moral emotions with relevance to sexual situations have been addressed in the literature: including fear, anger, disgust, and guilt.

Davis et al. (2023) argued that a person feeling such emotions as fear, anger or disgust during a sexual encounter might tend to view the behaviors of the other person as wrong in a way consistent with force or coercion: such as dangerous, unwanted, or otherwise consistent with coercion. Or an emotion such as shame or disgust can be associated with the view that the encounter is inappropriate for other reasons.

The emotion of disgust (both as a state or a trait), for example, has enjoyed considerable interest in the moral psychology literature and has been associated with a variety of moral judgments. Moreover, incidental triggers of disgust (unrelated to the issues to be judged) have also been shown to provoke harsher moral judgments, though the size of such effects is sometimes small (see; Haidt, 2012; Landy and Goodwin, 2015; Schnall et al., 2015; van Leeuwen et al., 2017 for reviews).

Sex can provoke disgust in any number of ways that don't involve coercion: such as sex with unattractive partners, specific sexual acts, sex with inappropriate partners, bodily fluids and smells, and more. As such, it has the potential to also provoke moral judgments involving coercion. Relevant to this, Haidt (2012) showed that when confronted with stories with themes of disgust and disrespect, but involving no harm to anyone, 38% of participants nevertheless claimed someone was harmed.

The moral psychology literature supplements the emotion priming literature in suggesting that emotions can infect judgments. It remains for future research to specifically investigate such effects specifically on judgments of consent and coercion.

### 7.1 Are “central details” of traumatic events really impervious to fading and distortion?

Some counterintuitive behavior experts have made claims regarding the fidelity of memory for trauma over time that could be viewed as controversial or problematic by memory researchers. For example, Dr. Jim Hopper's claim that “...memories of highly stressful or traumatic experiences, at least their most central details, don't tend to fade over time,” (Hopper, 2018b; see also van der Kolk, 1998). Researchers do agree that people tend to have stronger, longer lasting memories for emotional events, but also agree that all memories (even those for emotional/traumatic events) are subject to forgetting and will degrade over time (McNally et al., 2004; Laney and Loftus, 2005; Reisberg and Heuer, 2020). In fact, research has shown that people do forget the central and peripheral details of highly stressful/traumatic memories (Wagenaar and Groeneweg, 1990; Hirsh et al., 2011; see also research on vagaries of “flashbulb” memories for highly emotional events): (Rubin and Kozin, 1984; Christianson, 1989, 1992; Talarico and Rubin, 2003, 2007).

CBE Jim Hopper and others have also made claims regarding the imperviousness of traumatic memories to distortion. For example, while Dr. Hopper has conceded that the peripheral details of an event can easily be distorted, he also claimed that “...decades of research have shown that the most central details are not easy to distort, which typically requires repeated leading questions from people in authority or a very strong internal motivation for doing so” (Hopper, 2018b). Research has also soundly contradicted this claim. It is beyond our scope to offer a comprehensive review on this point, but the following are a few examples.

Since laboratory studies cannot create the level of distress experienced by victims of trauma, one way to address this claim is to look at changes in the reports of trauma victims, or those subject to highly stressful events, over time. Southwick et al. (1997), for example, examined the consistency of memories of combat-related traumatic events among veterans of Operation Desert Storm. Participants completed a questionnaire 1 month and at 2 years after returning from the war. Results showed that reports of 88% of participants changed over time. That is, 46% first reported traumatic events that they did not recall 2 years later and 70% recalled traumatic events at the 2-year evaluation that they did not report during the first evaluation. The researchers explained that many of these event memories that changed involved highly traumatic events that were specific and objective. For example, about 27% of participants changed their memory report for the event of “seeing others killed or wounded”. The researchers provided several explanations for the inconsistency in memory for these traumatic events, including the possibility that post-event information may have led to distortion. Though the mechanisms of change no doubt varied across persons, the results indicated that the presumed central details of a traumatic event (e.g., seeing others killed) are not indelible. Additionally, they provide presumptive evidence of the possibility that memory of traumatic events can be distorted in the absence of leading questions or other suggestive interviewing tactics. Similar changes in memory for highly emotional events have been documented in the previously referenced “flashbulb” memory research. People both forgot over time and changed memories for aspects of the events (Rubin and Kozin, 1984; Christianson, 1989, 1992; Talarico and Rubin, 2003, 2007).

Other studies have examined the effects of common sources of memory distortion on memories for highly stressful events. For example, Morgan et al. (2013) assessed the impact of misinformation on memories of military personnel in SERE (Survive, Evade, Resist, Escape) training, which has been shown to result in very high levels of stress and stress hormones. Participants had to survive in the wilderness, try to evade capture, endure capture and placement in a mock prisoner-of-war camp, undergo a stressful interrogation, and try to escape. The experience is meant to be realistic, and therefore, highly stressful. After the experience, participants completed a questionnaire with or without misinformation and leading questions. Results showed a significant influence of misinformation on participants' memory of the event: for example, 27% of participants in the misinformation condition falsely remembered a weapon, compared to only 3% of participants in the no misinformation condition. Participants were also asked to make an eyewitness identification of their interrogator in a target-absent photo array. Results showed that 91% of participants in the misinformation condition made a false-positive eyewitness identification, compared to 53% of participants in the no misinformation condition. While exposure to misleading information led to a significant increase in memory distortions compared to those who were not exposed to misinformation, more than half of the participants who did not receive misinformation still showed memory distortion in the form of false identifications. Memory for the central details of a highly stressful event was readily distorted by exposure to misleading information, even in this group of military personnel who are trained to withstand stress, propaganda, and other exploitation efforts.

These results are consistent with large bodies of research showing that memory for forensically important aspects of highly stressful events is subject to failure and distortion: such as memory for a perpetrator, war trauma experiences, and other traumatic event details (see McNally, 2003; Deffenbacher et al., 2004; Morgan et al., 2004, 2013). Moreover, there is no reason to believe that traumatic memories cannot be altered through the same processes that have been repeatedly shown to distort memories for countless real-life events: or to lead to memories of events that never happened at all (see McNally, 2003; Brainerd and Reyna, 2005; Davis and Loftus, 2007, 2020; Bialer et al., 2021).

### 7.2 Some foibles of “trauma-informed” interviewing

Our culture is replete with “trauma-informed” strategies of dealing with alleged victims of trauma (e.g., Reicherter et al., 2022). Among these are methods of interviewing alleged trauma victims that are intended to maximize the completeness and accuracy of their reports. Among the most commonly taught of these is “FETI,” or the “Forensic Experiential Trauma Interview” (Strand and Heitman, 2017).

Informed by the many researchers and educators on the “neurobiology of trauma,” the FETI method relies on the common claims that memories of trauma are fragmented, and not organized in the coherent sequence needed by those in the legal system: as reflected in our earlier quotes from Rebecca Campbell, one of the adopters of Strand's ideas.

Many other CBEs also express this opinion. Dr. David Lisak, a clinical psychologist and forensic consultant who researches the causes and consequences of interpersonal violence, and teaches the neurobiology of trauma to law enforcement, describes what happens when someone experiences a traumatic event as such: “When the amygdala responds to a life-threatening stimulus and reacts, we are no longer able to encode experiences in the same way. When the amygdala is firing due to something traumatizing, experiences get encoded as intense sensory fragments rather than coherent, sequential events” (Lisak, 2013). As a result, Dr. Lisak suggests, one should focus on asking the person what is remembered about these specific fragments, and should not ask for a sequential narrative. He does, at least, acknowledge that respondents will tend to try to cooperate and guess when asked about things they have not successfully encoded, and that any sequential narrative elicited by questions concerning sequence can be inaccurate.

https://www.youtube.com/watch?v=py0mVt2Z7nc

Still, the trauma-related fragmentation assumption itself seems to be flawed, in that the best research on the topic has revealed no differences in the fragmentation of memory for real life positive, vs. important, vs. traumatic events (see McNally, 2022 for review). If traumatic memories are actually no more fragmented than other memories, is it really necessary to have specialized interviewing procedures for trauma? Or should interviewing regarding all events be conducted using the same special procedures?

The claim of fragmentation of trauma memories, though not new (e.g., van der Kolk and Fisler, 1995), is now much more widespread: and recommended strategies of interviewing alleged trauma victims is based on the idea that memory for sensory fragments of the event will be accurate and should be the focus of questions. Trauma victims are presumed unable to have coherent sequential narratives and these should not be the (at least initial) focus of forensic interviews. Instead, trauma-informed interviewing strategies such as “FETI” suggest asking alleged victims about these kinds of fragments and peripheral details (sounds, smells, feelings, and so on) as a pathway of association that will lead to memory of litigation-relevant details. For example, John Reid Associates (arguably the most prominent interview/interrogation training organization in America), now trainers of FETI, recommends on their website that interviewers focus on seven questions: “What are you able to tell me about your experience?” “Tell me more about…(the room; the person; etc.).” “What was your thought process during this experience?” “What are you able to remember about…5 senses?” “What were your reactions to this experience.” “What is the most difficult part of this experience for you?” “What if anything can't you forget about your experience.” While it is true that some details elicited in this manner can lead through associative pathways to other relevant information, they are nevertheless risky in some respects.

First, the account elicited through such questions is highly likely to be fragmented and disorganized, seeming to confirm the testimony of CBEs concerning the nature of trauma memories, and inviting jurors to believe that because the account is fragmented the victim was traumatized (and impliedly the event was indeed rape). Though CBEs are quite right in noting the impact of the way trauma victims are interviewed on the coherence of the narratives elicited, there are other ways of questioning that increase coherence without suggestion. For example, consistent with the principles of cognitive interviewing (Fisher and Geiselman, 1992, 2010), Taylor and colleagues showed that simply asking the person to describe their experience results in a more coherent narrative than use of a series of specific questions regarding details of sequence, persons, context, or events (Taylor et al., 2020). Though FETI does incorporate the relatively open-ended “tell me about” question types of the cognitive interview, it also imposes a different structure by directing attention firmly away from narrative structure to unorganized fragments.

The phenomenon of “retrieval-induced forgetting” (see Bäuml and Kliegl, 2017 for a review), whereby selective retrieval (and particularly repeated retrieval) of some aspects of an event can lead to greater forgetting of others, would also suggest that emphasis on retrieval of non-crucial details might be unwise, particularly when repeated.

More relevant to the focus of this review is the issue of how the procedure of asking the alleged victim to focus on feelings and emotions (and even other sensory details) can serve to prime those emotions and serve as context for recall. As Davis et al. have pointed out elsewhere (Davis and Loftus, 2016, 2019; Davis et al., 2023), emotion can serve similar biasing functions at recall to those at encoding (see also Bower and Forgas, 2001; Forgas, 2008; Gibbons et al., 2018).

First, the emotions felt at retrieval are not necessarily the same as those present at encoding (e.g., Levine et al., 2006; Schmidt et al., 2021). This can happen for at least two reasons. The emotions felt during a sexual encounter might not have been so negative as they later became. Clancy (2011) documented this with many sexual abuse victims who did not begin to feel traumatized by their abuse until they were old enough to understand what it was and why it was so inappropriate. This is also discussed in literature on “unacknowledged rape,” regarding those who initially did not regard themselves as victims, but later came to “understand” that they had actually been raped (e.g., Kahn and Mathie, 2000; Wilson and Miller, 2016). Finally, appraisal theorists (e.g., Schmidt et al., 2010) have suggested that biases in memory for emotion can be explained by how much significance a past event is given by the person in the present (e.g., Scherer et al., 2001), and that this might explain some findings that people tend to overestimate the negativity of past emotions (e.g., Schrader et al., 1990; Bryant, 1993; Parkinson et al., 1995; Cutler et al., 1996; Barrett, 1997; Safer et al., 2001; Lench and Levine, 2010; Levine et al., 2021). Moreover, it poses the potential that current negative emotions might provoke a more negative view of a past sexual encounter than warranted by the behaviors at the time.

The emotions a person relies upon for recall can also become different than those experienced at encoding because when the person is asked to describe the emotions they experienced during the event at a later time, they might misremember them. A substantial literature has documented inconsistency in recall of emotions across time. Memory for emotion can be distorted by the same processes as can other memories. Emotion memories can also become distorted to serve current motivations or goals, and to be consistent with current beliefs about oneself and other issues. There is also a tendency toward recalling one's own autobiography and experiences as consistent with current feelings and views of oneself. These and other processes undermine the consistency of emotion between then and now (see Levine et al., 2009; Davis and Loftus, 2019 for reviews). Thus, the emotion relied on for retrieval might both misdirect retrieval of relevant facts, and also bias memory toward consistency with it, as discussed next.

Second, whereas emotion can direct attention during encoding, it can also direct retrieval, leading the person to selectively recall emotion-consistent information as well as to interpret it in an emotion-consistent manner (as incorporated in the previously referenced AIM model: Forgas, 2002). Judgments at recall can then be biased because they are based on incomplete information, the set of which is consistent with the emotion, even though emotion-inconsistent information might have originally predominated.

Third, given that many sexual assaults are not reported immediately, it is important to note that as memories become more vague with time the potential for distortion increases. As suggested by “fuzzy trace” theory (e.g., Brainerd and Reyna, 2005; Brainerd et al., 2022), when verbatim memories are vague, strong emotion memories can lead to constructive memory errors consistent with the emotion and how it would likely be produced. If FETI theorizing is correct regarding the vagueness of trauma memories, this leaves open greater opportunity for memory distortion in the direction of expectations triggered by emotion and other remembered details of context, as well as expectations based on one's self-concept and other general knowledge that tends to infect “fuzzy” memories. As Davis and colleagues (Davis and Loftus, 2016, 2019; Davis et al., 2023) have pointed out, and as discussed earlier regarding effect of emotion on encoding, knowledge and expectations tend to include those regarding circumstances likely to produce the emotions in question: such as when fear or disgust during a sexual encounter will be viewed as consistent with coercion or rape. Given the above considerations, the wisdom of selective priming of emotions and sensory fragments during retrieval, and of relying on the accuracy of resulting reports is questionable.

## 8 Conclusions

“Extraordinary Claims Require Extraordinary Evidence”

Carl Sagan

What has become known as “the Sagan standard,” or “ECREE” seems particularly relevant to the claims of CBEs regarding traumatic memories (Sagan, 1979; Sagan and Druyan, 1997). The claims that trauma causes the brain functions underlying memory to operate in fundamentally different ways than those underlying memories for less stressful events truly are extraordinary: as are those that traumatic memories that are laid down are unerringly accurate, do not fade over time, and are resistant or impervious to distortion. As Sagan and others have pointed out, the burden of proof for such extraordinary claims is on the claimant, not on those who doubt it. Yet, what extraordinary evidence has been offered for such claims regarding trauma memories? We suggest that there is no extraordinary evidence in support of the claims of CBEs regarding trauma memory, where there is, as we have documented herein, significant evidence to contradict them.

## Author contributions

DD: Writing – original draft. AH: Writing – original draft. DH: Writing – original draft.

## Footnotes

^1^A full review of the literature of effects of stress on memory is beyond our scope. However, it is important to note that this literature is not fully consistent in showing either negative effects of negative emotion (or stress) on accuracy (most prominently shown in the eyewitness memory literature) vs. positive effects [most prominently shown in the basic memory literature: see review by Marr et al. (2021)]. Most importantly, however, many studies have created stress that is independent of the to-be-remembered event (e.g., exposure to the cold pressor task before or during the stimulus materials or event. Research that would be relevant to our current discussion would involve strong emotions produced by the event to be remembered, such as is most often the case in the eyewitness memory literature, and is rarely the case in the general memory literature (see Marr et al., 2021). It would also involve events, rather than face memory or memory for pictures or words (which is common in neither literature), and assessment of memory for aspects of the event with potential for emotion-consistent distortion (also common in neither literature). As such, little of the basic literature on stress and witness memory is directly relevant to our discussion. Therefore, we focus largely on literature on emotion and interpretation: which is directly relevant to what is encoded into memory.

## References

[B1] Adams-Curtis L. E. Forbes G. B. (2004). College women's experiences of sexual coercion: a review of cultural, perpetrator, victim, and situational variables. Trauma Violence Abuse 5, 91–122. 10.1177/1524838003262331 15070552

[B2] Ariely D. Loewenstein G. F. (2006). The heat of the moment: the effect of sexual arousal on sexual decision making. J. Behav. Decis. Mak. 19, 87–98. 10.1002/bdm.50120862534

[B3] Avramova Y. R. Inbar Y. (2013). Emotion and moral judgment. Cogn. Sci. 4, 169–178. 10.1002/wcs.1216 26304193

[B4] Bargh J. A. Raymond P. (1995). The naive misuse of power: nonconscious sources of sexual harassment. J. Soc. Issues 51, 85–96. 10.1111/j.1540-4560.1995.tb01310.x

[B5] Bargh J. A. Raymond P. Pryor J. B. Strack F. (1995). Attractiveness of the underling: an automatic power → sex association and its consequences for sexual harassment and aggression. J. Pers. Soc. Psychol. 68, 768–781. 10.1037/0022-3514.68.5.768 7776181

[B6] Barrett L. F. (1997). The relationships among momentary emotion experiences, personality descriptions, and retrospective ratings of emotion. Pers. Soc. Psychol. Bull. 23, 1100–1110. 10.1177/01461672972310010

[B7] Bäuml K.-H. T. Kliegl O. (2017). “Retrieval-induced remembering and forgetting,” in Learning and Memory: A Comprehensive Reference, 2nd Edn, Vol. 2, ed J. H. Byrne (San Diego, CA: Academic Press), 27–51.

[B8] Bialer D. M. Reyna V. F. Brainerd C. J. (2021) “False memory: what are the effects, how does fuzzy-trace theory predict them, how does this matter for eyewitness testimony?,” in Methods, Measures, Theories in Eyewitness Identification Tasks, eds A. M. Smith, M. Toglia, J. M. Lampinen (New York, NY: Routledge/Taylor & Francis Group), 325–352

[B9] Blanton H. Gerrard M. (1997). Effect of sexual motivation on men's risk perception for sexually transmitted disease: there must be 50 ways to justify a lover. Health Psychol. 16:374. 10.1037/0278-6133.16.4.374 9237090

[B10] Bohner G. Eyssel F. Pina A. Siebler F. Viki G. T. (2009). “Rape myth acceptance: cognitive, affective and behavioural effects of beliefs that blame the victim and exonerate the perpetrator,” in Rape: Challenging Contemporary Thinking, 1st Edn, eds. M. Horvath and J. Brown (Uffculme: Willan Publishing, 17–45.

[B11] Bonsall M. B. Holmes E. A. (2023). Temporal dynamics of trauma memory persistence. J. R. Soc. Interface 20:20230108. 10.1098/rsif.2023.0108 37282590 PMC10244962

[B12] Bouffard J. A. Miller H. A. (2014). The role of sexual arousal and overperception of sexual intent within the decision to engage in sexual coercion. J. Interpers. Violence 29, 1967–1986. 10.1177/0886260513515950 24407143

[B13] Bower G. H. Forgas J. P. (2001). “Mood and social memory,” in Handbook of Affect and Social Cognition, ed J. P.Forgas (Mahwah, NJ: Lawrence Erlbaum Associates), 95–120.

[B14] Brainerd C. J. Bialer D. M. Chang M. (2022). Fuzzy-trace theory and false memory: meta-analysis of conjoint recognition. J. Exp. Psychol. Learn. Mem. Cogn. 48, 1680–1697. 10.1037/xlm0001040 34516208

[B15] Brainerd C. J. Chang M. Bialer D. M. (2021). Emotional ambiguity and memory. J. Exp. Psychol. Gen. 150, 1476–1499. 10.1037/xge0001011 33332144

[B16] Brainerd C. J. Reyna V. F. (2005). The Science of False Memory. Oxford University Press.

[B17] Brainerd C. J. Reyna V. F. (2019). Fuzzy-trace theory, false memory, and the law. Policy Insights Behav. Brain Sci. 6, 79–86. 10.1177/2372732218797143

[B18] Brainerd C. J. Stein L. M. Silveira R. A. Rohenkohl G. Reyna V. F. (2008). How does negative emotion cause false memories? Psychol. Sci. 19, 919–925. 10.1111/j.1467-9280.2008.02177 18947358

[B19] Brokke S. S. Bertelsen T. B. Landrø N. I. Haaland V. Ø. (2022). The effect of sexual abuse and dissociation on suicide attempt. BMC Psychiatry 22:29. 10.1186/s12888-021-03662-9 35012509 PMC8751353

[B20] Brosch T. Scherer K. R. Grandjean D. Sander D. (2013). The impact of emotion on perception, attention, memory, and decision-making. Swiss Med. Wkly. 143, 1–10. 10.4414/smw.2013.13786 23740562

[B21] Bryant R. A. (1993). Memory for pain and affect in chronic pain patients. Pain 54, 347–351. 10.1016/0304-3959(93)90036-O 8233551

[B22] Caamano-Isorna F. Adkins A. Moure-Rodriguez L. Conley A. H. Dick D. (2021). Alcohol use and sexual and physical assault victimization among university students: three years of follow-up. J. Interpers. Viol. 36, NP3574–NP3595. 10.1177/0886260518780413 29897019 PMC6699923

[B23] Campbell R. (2012). The Neurobiology of Sexual Assault: Implications for Law Enforcement, Prosecution and Victim Advocacy. Research for the Real. Available online at: https://nij.ojp.gov/media/video/24056#transcript−0 (accessed June 26, 2024).

[B24] Campbell R. (2014). Sexual assault: A trauma informed to law enforcement first response, part 1 [Video]. YouTube. Available online at: https://www.youtube.com/watch?v=CnlXzD2pYSA (accessed June 26, 2024).

[B25] Chang V. T. Overall N. C. Madden H. Low R. S. T. (2018). Expressive suppression tendencies, projection bias in memory of negative emotions, and well-being. Emotion 18, 925–941. 10.1037/emo0000405 29389201

[B26] Christianson S. Å. (1992). Emotional stress and eyewitness memory: a critical review. Psychol. Bull. 112:284. 10.1037/0033-2909.112.2.284 1454896

[B27] Christianson S. Á. (1989). Flashbulb memories: special, but not so special. Mem. Cogn. 17, 435–443. 10.3758/BF03202615 2761401

[B28] Clancy S. A. (2011). The Trauma Myth: The Truth About the Sexual Abuse of Children—anD its Aftermath. New York, NY: Basic Books.

[B29] Clore G. L. Schiller A. J. Shaked A. (2018). Affect and cognition: three principles. Curr. Opin. Behav. Sci. 19, 78–82. 10.1016/j.cobeha.2017.11.010 30271831 PMC6159898

[B30] Clore G. L. Schnall S. (2019). “The influence of affect on attitude,” in The Handbook of Attitudes, Volume I: Basic Principles, 2nd Edn, eds. D. Albarracín and B. T. Johnson (Routledge), 259–290.

[B31] Coane J. H. (2013). Retrieval practice and elaborative encoding benefit memory in younger and older adults. J. Appl. Res. Mem. Cogn. 2, 95–100. 10.1016/j.jarmac.2013.04.001

[B32] Cutler S. E. Larson R. J. Bunce S. C. (1996). Repressive coping style and the experience and recall of emotion: a naturalistic study of daily affect. J. Pers. 64, 379–405. 10.1111/j.1467-6494.1996.tb00515.x 8656322

[B33] Davis D. Cano J. Miller G. Loftus E. F. (2023). The multiple roles of emotion in memory for disputed sexual encounters. Top. Cogn. Sci. 1–17. 10.1111/tops.12691 37801703

[B34] Davis D. Loftus E. F. (2004). “What's good for the goose cooks the gander: inconsistencies between the law and psychology of voluntary intoxication and sexual assault,” in Handbook of Forensic Psychology: Resource for Mental Health and Legal Professionals, eds. W. T. O'Donohue and E. R. Levensky (Cambridge, MA: Academic Press), 997–1032.

[B35] Davis D. Loftus E. F. (2007). “Internal and external sources of distortion in adult witness memory,” in Handbook of Eyewitness Memory, Vol. 1: Memory for Events, eds M. P. Toglia, J. D. Read, D. R. Ross, and R. C. L. Lindsay (Mahwah, NJ: Erlbaum), 195–237.

[B36] Davis D. Loftus E. F. (2009). “Expectancies, emotion, and memory reports for visual events,” in The Visual World in Memory, 1st Edn, ed. J. Brockmole (London: Psychology Press), 178–214.

[B37] Davis D. Loftus E. F. (2016). Remembering disputed sexual encounters: A new frontier for witness memory research. J. Crim. L. Criminol. 105:811.

[B38] Davis D. Loftus E. F. (2019). Title IX and “Trauma-Focused” investigations: the good, the bad and the ugly. J. Appl. Res. Mem. Cogn. 8, 403–410. 10.1016/j.jarmac.2019.08.001

[B39] Davis D. Loftus E. F. (2020). “Recovered memories and false memories,” in New Oxford Textbook of Psychiatry, 3rd Edn, eds N. Andreasen, J. Geddes, and G. Goodwin (Oxford: Oxford University Press).

[B40] Deffenbacher K. A. Bornstein B. H. Penrod S. D. McGorty E. K. (2004). A meta-analytic review of the effects of high stress on eyewitness memory. Law Hum. Behav. 28, 687–706. 10.1007/s10979-004-0565-x 15732653

[B41] Ditto P. H. Pizarro D. A. Epstein E. B. Jacobson J. A. MacDonald T. K. (2006). Visceral influences on risk taking behavior. J. Behav. Decis. Mak. 19, 99–113. 10.1002/bdm.520

[B42] Easterbrook J. A. (1959). The effect of emotion on cue utilization and the organization of behavior. Psychol. Rev. 66, 183–201. 10.1037/h0047707 13658305

[B43] Fenner L. (2017). Sexual consent as a scientific subject: a literature review. Am. J. Sex. Educ. 12, 451–471. 10.1080/15546128.2017.1393646

[B44] Fisher R. P. Geiselman R. E. (1992). Memory-Enhancing Techniques for Investigative Interviewing: The Cognitive Interview. Springfield: Charles C Thomas Publisher.

[B45] Fisher R. P. Geiselman R. E. (2010). The cognitive interview method of conducting police interviews: eliciting extensive information and promoting therapeutic jurisprudence. Int. J. Law Psychiatry 33, 321–328. 10.1016/j.ijlp.2010.09.004 20875685

[B46] Fiske S. T. (1993). Controlling other people: the impact of power on stereotyping. Am. Psychol. 48, 621–628. 10.1037/0003-066X.48.6.621 8328729

[B47] Forgas J. P. (2002). Feeling and doing: affective influences on interpersonal behavior. Psychol. Inq. 13, 1–28. 10.1207/S15327965PLI1301_01

[B48] Forgas J. P. (2008). “Affect, cognition, and social behavior: the effects of mood on memory, social judgments, and social interaction,” in Memory and Mind: A Festschrift for Gordon H. Bower, eds M. A. Gluck, J. R. Anderson, and S. M. Kosslyn (Mahwah, NJ: Lawrence Erlbaum Associates), 261–279.

[B49] Fredrickson B. L. (2000). Extracting meaning from past affective experiences: the importance of peaks, ends, and specific emotions. Cogn. Emot. 14, 577–606. 10.1080/026999300402808

[B50] Galinsky A. D. Gruenfeld D. H. Magee J. C. (2003). From power to action. J. Pers. Soc. Psychol. 85, 453–466. 10.1037/0022-3514.85.3.453 14498782

[B51] Galinsky A. D. Magee J. C. Gruenfeld D. H Whitson J. A. Liljenquist K. A. (2008). Power reduces the press of the situation: implications for creativity, conformity, and dissonance. J. Pers. Soc. Psychol. 95, 1450–1466. 10.1037/a0012633 19025295

[B52] Galinsky A. D. Rucker D. D. Magee J. C. (2016). Power and perspective-taking: a critical examination. J. Exp. Soc. Psychol. 67, 91–92. 10.1016/j.jesp.2015.12.002

[B53] Gibbons H. Seib-Pfeifer L.-E. Koppelheie-Gossel J. Schnuerch R. (2018). Affective priming and cognitive load: event-related potentials suggest and interplay of implicit affect misattribution and strategic inhibition. Psychophysiology 55, 1–19. 10.1111/psyp.13009 28940207

[B54] Greenwald A. G. Banaji M. R. (2017). The implicit revolution: reconceiving the relation between conscious and unconscious. Am. Psychol. 72:861. 10.1037/amp0000238 29283625

[B55] Greifeneder R. Bless H. Pham M. T. (2011). When do people rely on affective and cognitive feelings in judgment? A review. Pers. Soc. Psychol. Rev. 15, 107–141. 10.1177/1088868310367640 20495111

[B56] Guinote A. (2007). Power and goal pursuit. Pers. Soc. Psychol. Bull. 33, 1076–1087. 10.1177/0146167207301011 17575244

[B57] Guinote A. E. (2010). In touch with your feelings: power increases reliance on bodily information. Soc. Cogn. 28, 110–121. 10.1521/soco.2010.28.1.110

[B58] Guinote A. E. Vescio T. K. (2010). The Social Psychology of Power. New York, NY: Guilford Press.

[B59] Haidt J. (2001). The emotional dog and its rational tail: a social intuitionist approach to moral judgment. Psychol. Rev. 108, 814–834. 10.1037/0033-295X.108.4.814 11699120

[B60] Haidt J. (2012). The Righteous Mind: Why Good People are Divided by Politics and Religion. New York, NY: Pantheon.

[B61] Harmon-Jones E. Gable P. A. Price T. F. (2013). Does negative affect always narrow and positive affect always broaden the mind? Considering the influence of motivational intensity on cognitive scope. Curr. Dir. Psychol. Sci. 22, 301–307. 10.1177/0963721413481353

[B62] Hirsh J. B. Galinsky A. D. Zhong C. B. (2011). Drunk, powerful, and in the dark: how general processes of disinhibition produce both prosocial and antisocial behavior. Perspect. Psychol. Sci. 6, 415–427. 10.1177/1745691611416992 26168194

[B63] Hoffman D. D. (2019). The Case Against Reality: Why Evolution Hid the Truth From Our Eyes. New York, NY: W.W. Norton & Company.

[B64] Hogan A. Hart D. Miller G. Davis D. (2024). Perceptions of Sexually Coercive Behaviors [Poster presentation]. San Francisco, CA: The Association for Psychological Science Annual Convention.

[B65] Hopper J. (2015). Scientific Research. Available online at: https://jimhopper.com/topics/child-~abuse/recovered-memories-of-sexual-abuse/scientific-research/ (accessed June 26, 2024).

[B66] Hopper J. (2018a). How Reliable Are the Memories of Sexual Assault Victims? Scientific American. Available online at: https://blogs.scientificamerican.com/observations/how-reliable-are-the-memories-of-sexual-assault-victims/ (accessed June 26, 2024).

[B67] Hopper J. (2018b). Sexual Assault & the Brain in Six Minutes. Available online at: https://www.youtube.com/watch?v=7G8weF4jsZw&t=1s (accessed June 26, 2024).

[B68] Hopper J. (2023). Recovered Memories of Sexual Abuse. Available online at: https://jimhopper.com/topics/child-abuse/recovered-memories-of-sexual-abuse/ (accessed June 26, 2024).

[B69] Hopper J. (n.d.-b). Potential Overlaps of Dissociation with Freezing, Habit Behaviors, and/or Extreme Survival Reflexes [PowerPoint slide]. Available online at: https://jimhopper.com/pdf/hopper_dissociation_overlaps.pdf.

[B70] Hopper J. Lisak D. (2014). Why rape and trauma survivors have fragmented and incomplete memories. Time Magazine. Available online at: https://time.com/3625414/rape-trauma-brain-memory/

[B71] Huntsinger J. R. (2012). Does positive affect broaden and negative affect narrow attentional scope? A new answer to an old question. J. Exp. Psychol. Gen. 141, 595–600. 10.1037/a0027709 22409665

[B72] Huntsinger J. R. (2013). Does emotion directly tune the scope of attention? Curr. Dir. Psychol. Sci. 22, 265–270. 10.1177/0963721413480364

[B73] Imhoff R. Schmidt A. F. (2014). Sexual disinhibition under sexual arousal: Evidence for domain specificity in men and women. Arch. Sex. Behav. 43, 1123–1136. 10.1007/s10508-014-0329-8 25091213

[B74] Kabota S. Nakazawa E. (2022). Concept and implications of sexual consent for education: a systematic review of empirical studies. Sex. Relat. Therapy 1–23. 10.1080/14681994.2022.2039617

[B75] Kahn A. S. Mathie V. A. (2000). “Understanding the unacknowledged rape victim,” in Sexuality, Society, and Feminism, 1st Edn, eds. C. B. Travis and J. W. White (Washington, DC: American Psychological Association), 377–403. 10.1037/10345-015

[B76] Kahneman D. (2011). Thinking, Fast and Slow. Macmillan. New York, NY: Farrar, Straus, & Giroux.

[B77] Kaplan R. L. Van Damme I. Levine L. J. (2012). Motivation matters: differing effects of pre-goal and post-goal emotions on attention and memory. Front. Psychol. 3:404. 10.3389/fpsyg.2012.00404 23162490 PMC3498897

[B78] Keltner D. Gruenfeld D. H. Anderson C. (2003). Power, approach, and inhibition. Psychol. Rev. 110:265. 10.1037/0033-295X.110.2.265 12747524

[B79] Kern R. P. Libkuman T. M. Otani H. Holmes K. (2005). Emotional stimuli, divided attention, and memory. Emotion 5, 408–417. 10.1037/1528-3542.5.4.408 16366745

[B80] Kim B. K. Zauberman G. (2013). Can Victoria's Secret change the future? A subjective time perception account of sexual-cue effects on impatience. J. Exp. Psychol. Gen. 142, 328–335. 10.1037/a0028954 22686639

[B81] Kindelan K. (2018). Rose McGowan's Account of Detaching From Reality During Alleged Rape Is Common: Experts. Good Morning America. Available online at:https://www.goodmorningamerica.com/wellness/story/rose-mcgowans-account-~detaching-reality-alleged-rape-common-52747906 (accessed June 26, 2024).

[B82] Kunstman J. W. Maner J. K. (2011). Sexual overperception: Power, mating motives, and biases in social judgment. J. Pers. Soc. Psychol. 100:282. 10.1037/a0021135 21142379

[B83] Lammers J. Galinsky A. D. Dubois D. Rucker D. D. (2015). Power and morality. Curr. Opin. Psychol. 6, 15–19. 10.1016/j.copsyc.2015.03.018

[B84] Lammers J. Galinsky A. D. Gordijn E. H. Otten S. (2008). Illegitimacy moderates the effects of power on approach. Psychol. Sci. 19, 558–564. 10.1111/j.1467-9280.2008.02123.x 18578845

[B85] Lammers J. Imhoff R. (2016). Power and sadomasochism: understanding the antecedents of a knotty relationship. Soc. Psychol. Personal. Sci. 7, 142–148. 10.1177/1948550615604452

[B86] Lammers J. Stoker J. I. Jordan J. Pollmann M. Stapel D. A. (2011). Power increases infidelity among men and women. Psychol. Sci. 22, 1191–1197. 10.1177/0956797611416252 21771963

[B87] Landy J. F. Goodwin G. P. (2015). Does incidental disgust amplify moral judgment? A meta-analytic review of experimental evidence. Perspect. Psychol. Sci. 10, 518–536. 10.1177/17456916155831 26177951

[B88] Laney C. Loftus E. F. (2005). Traumatic memories are not necessarily accurate memories. Can. J. Psychiatry 50, 823–828. 10.1177/070674370505001303 16483115

[B89] Lee H. Roh S. Kim D. J. (2009). Alcohol-induced blackout. Int. J. Environ. Res. Public Health 6, 2783–2792. 10.3390/ijerph6112783 20049223 PMC2800062

[B90] Lench H. C. Levine L. J. (2010). Motivational biases in memory for emotions. Cogn. Emot. 24, 401–418. 10.1080/02699930802650788

[B91] Levine L. J. Edelstein R. S. (2009). Emotion and memory narrowing: a review and goal-relevance approach. Cogn. Emot. 23, 833–875. 10.1080/02699930902738863

[B92] Levine L. J. Lench H. C. Safer M. A. (2009). Functions of remembering and misremembering emotion. Appl. Cogn. Psychol. 23, 1059–1075. 10.1002/acp.1610

[B93] Levine L. J. Murphy G. Lench H. C. Greene C. M. Loftus E. F. Tinti C.. (2021). Remembering facts versus feelings in the wake of political events. Cogn. Emot. 35, 936–955. 10.1080/02699931.2021.1910496 33829942

[B94] Levine L. J. Pizarro D. A. (2004). Emotion and memory research: a grumpy overview. Soc. Cognit. 22(5: Special issue), 530–554. 10.1521/soco.22.5.530.50767

[B95] Levine L. J. Safer M. A. Lench H. C. (2006). “Remembering and misremembering emotions,” in Judgments Over Time: The Interplay of Thoughts, Feelings, and Behaviors, eds. L. J. Sanna, and E. C. Chang (Oxford: Oxford University Press), 271–290.

[B96] Lisak D. (2013). Neurobiology of trauma - Dr. David Lisak [Video]. [ACASA Arkansas]. YouTube. Available online at: https://www.youtube.com/watch?v=py0mVt2Z7nc (accessed June 26, 2024).

[B97] Livingston T. N. Davis D. (2020). Power affects perceptions of sexual willingness: Implications for litigating sexual assault allegations. Viol. Gender 7, 116–120. 10.1089/vio.2019.0068

[B98] Loewenstein G. (1996). Out of control: visceral influences on behavior. Org. Behav. Hum. Decis. Process 65, 272–292. 10.1006/obhd.1996.0028

[B99] Loftus E. F. Loftus G. R. Messo J. (1987). Some facts about “weapon focus.” Law Hum. Behav. 11, 55–62. 10.1007/BF01044839

[B100] Lord C. G. Taylor C. A. (2009). Biased assimilation: effects of assumptions and expectations on the interpretation of new evidence: biased assimilation. Soc. Personal. Psychol. Compass 3, 827–841. 10.1111/j.1751-9004.2009.00203.x

[B101] Lynch S. Weber S. Kaplan S. Craun E. (2023). Childhood and adult sexual violence exposures as predictors of PTSD, dissociation, and substance use in women in jail. J. Child Sex. Abuse 1–17. 10.1080/10538712.2023.2226132 37357921

[B102] Mackworth N. H. (1965). Visual noise causes tunnel vision. Psychon. Sci. 3, 67–68. 10.3758/BF03343023

[B103] Magee J. C. Smith P. K. (2013). The social distance theory of power. Person. Soc. Psychol. Rev. 17, 158–186. 10.1177/1088868312472732 23348983

[B104] Maner J. K. Kendrick D. T. Neuberg S. L. Becker D. V. Robertson T. Hofer B.. (2005). Functional projection: how fundamental social motives can bias interpersonal perception. J. Pers. Soc. Psychol. 88, 63–78. 10.1037/0022-3514.88.1.63 15631575

[B105] Marr C. Sauerland M. Otgaar H. Quaedflieg C. W. E. M. Hope L. (2021). The effects of acute stress on eyewitness memory: an integrative review for eyewitness researchers. Memory 20, 1091–1100. 10.1080/09658211.2021.1955935 34309476

[B106] McAuliffe W. H. (2019). Do emotions play an essential role in moral judgments? Think. Reason. 25, 207–230. 10.1080/13546783.2018.1499552

[B107] McNally R. J. (2003). Remembering Trauma. Cambridge, MA: The Belknap Press of Harvard University Press.

[B108] McNally R. J. (2022). Are memories of sexual assault fragmented? Memory 30, 26–30. 10.1080/09658211.2020.1871023 33435857

[B109] McNally R. J. Clancy S. A. Barrett H. M. (2004). “Forgetting trauma?” in Memory and Emotion, eds. D. Reisberg and P. Hertel (Oxford University Press), 129–154. 10.1093/acprof:oso/9780195158564.003.000436389024

[B110] Melaragno E. (2018). Trauma in the body: An interview with Dr. Bessel van der Kolk. Still Harbor. Available online at: https://www.dailygood.org/story/1901/trauma-in-the-body-an-interview-with-dr-bessel-van-der-kolk-elissa-melaragno/ (accessed June 26, 2024).

[B111] Miller G. Davis D. (2024). Effects of male physical attractiveness and career potential on ratings of implications of female behaviors for sexual willingness (Unpublished Manuscript).

[B112] Mitchell J. (2023). Emotion and attention. Philos. Stud. 180, 73–99. 10.1007/s11098-022-01876-5

[B113] Morgan C. A. III. Hazlett G. Doran A. Garrett S. Hoyt G. Thomas P.. (2004). Accuracy of eyewitness memory for persons encountered during exposure to highly intense stress. Int. J. Law Psychiatry 27, 265–279. 10.1016/j.ijlp.2004.03.004 15177994

[B114] Morgan C. A. III. Southwick S. Steffian G. Hazlett G. A. Loftus E. F. (2013). Misinformation can influence memory for recently experienced, highly stressful events. Int. J. Law Psychiatry 36, 11–17. 10.1016/j.ijlp.2012.11.002 23219699

[B115] Muehlenhard C. Humphreys T. Jozkowski K. Peterson J. (2016). The complexities of sexual consent among college students: a conceptual and empirical review. J. Sex Res. 53, 1–31. 10.1080/00224499.2016.1146651 27044475

[B116] Muehlenhard C. L. Hollabaugh L. C. (1988). Do women sometimes say no when they mean yes? The prevalence and correlates of women's token resistance to sex. J. Pers. Soc. Psychol. 54, 872–879. 10.1037/0022-3514.54.5.872 3379584

[B117] Murray D. R. Murphy S. C. von Hippel W. Trivers R. Haselton M. G. (2017). A preregistered study of competing predictions suggests that men do overestimate women's sexual intent. Psychol. Sci. 28, 253–255. 10.1177/0956797616675474 28056212

[B118] Parkinson B. Briner R. B. Reynolds S. Totterdell P. (1995). Time frames for mood: relations between momentary and generalized ratings of affect. Pers. Soc. Psychol. Bull. 21, 331–339. 10.1177/0146167295214003

[B119] People v. Mayberry (1975). 15 Cal.3d 143. SCOCAL. Available online at: https://scocal.stanford.edu/opinion/people-v-mayberry-23014 (accessed June 26, 2024).

[B120] Percy J. (2023). What People Misunderstand About Rape. The New York Times. Available online at: https://www.nytimes.com/2023/08/22/magazine/immobility-rape-trauma-freeze.html#:~:text=Sexual%20assault%20often%20goes%20unpunished,an%20involuntary%20response%20to%20trauma (accessed June 26, 2024).

[B121] Pickel K. (1999). The influence of context on the “weapon focus” effect. Law Hum. Behav. 23, 299–311. 10.1023/A:1022356431375

[B122] Pike B. E. Galinsky A. D. (2020). Power leads to action because it releases the psychological brakes on action. Curr. Opin. Psychol. 33, 91–94. 10.1016/j.copsyc.2019.06.028 31404768

[B123] Pryor J. B. Stoller L. M. (1994). Sexual cognition processes in men high in the likelihood to sexually harass. Pers. Soc. Psychol. Bull. 20, 163–169. 10.1177/0146167294202003

[B124] Reicherter D. Wang, S. Ohrtman T. H. Ndukwe N. Vaatainen S. Alcalay S. Brown L. M. (2022). Implementation of trauma-informed best practices for international criminal investigations conducted by the United Nations Investigative Team to promote accountability for crimes committed by Da'esh/ISIL (UNITAD). Psychol. Inj. Law 15, 319–329. 10.1007/s12207-022-09457-x

[B125] Reisberg D. Heuer F. (2020). “Emotion's (varied) impact on memory for sexual misconduct,” in Memory and Sexual Misconduct: Psychological Research for Criminal Justice, eds. J. Pozzulo, E. Pica, and C. Sheahan (New York, NY: Routledge; Taylor & Francis Group), 7–41. 10.4324/9780429027857-2

[B126] Rerick P.O. Livingston T. N. Davis D. (2020). Does the horny man think women want him too? Effects of male sexual arousal on perceptions of female willingness. J. Soc. Psychol. 160, 520–533. 10.1080/00224545.2019.1692330 31722652

[B127] Rerick P. O. Livingston T. N. (2022). Beauty is in the eye of the beholder, and so is intent: men's interpretations of the sexual intent of attractive versus unattractive women. Violence Gend. 9, 193–200. 10.1089/vio.2022.0014

[B128] Rerick P. O. Livingston T. N. Davis D. (2019). “Rape and the jury,” in Handbook of Sexual Assault and Sexual Assault Prevention, eds. W. O. O'Donohue, C. Cummings, and P. Schewe (Springer), 551–571. 10.1007/978-3-030-23645-8_33

[B129] Rerick P. O. Livingston T. N. Davis D. (2022). Let's just do it: Sexual arousal's effects on attitudes regarding sexual consent. J. Soc. Psychol. 164, 456–472. 10.1080/00224545.2022.2106174 35924697

[B130] Richards J. M. Gross J. J. (1999). Composure at any cost? The cognitive consequences of emotion suppression. Pers. Soc. Psychol. Bull. 25, 1033–1044. 10.1177/014616729925110

[B131] Richards J. M. Gross J. J. (2000). Emotion regulation and memory: the cognitive costs of keeping one's cool. J. Pers. Soc. Psychol. 79:410. 10.1037/0022-3514.79.3.410 10981843

[B132] Richards J. M. Gross J. J. (2006). Personality and emotional memory: how regulating emotion impairs memory for emotional events. J. Res. Pers. 40, 631–651. 10.1016/j.jrp.2005.07.002

[B133] Rubin D. C. Kozin M. (1984). Vivid memories. Cognition 16, 81–95. 10.1016/0010-0277(84)90037-4 6540650

[B134] Ryan K. (2011). The relationship between rape myths and sexual scripts: the social construction of rape. Sex Roles 65, 774–782. 10.1007/s11199-011-0033-2

[B135] Safer M. A. Bonanno G. A. Field N. P. (2001). “It was never that bad”: biased recall of grief and long-term adjustment to the death of a spouse. Memory 9, 195–203. 10.1080/09658210143000065 11469313

[B136] Safer M. A. Christianson S.-Å. Autry M. W. Österlund K. (1998). Tunnel memory for traumatic events. Appl. Cogn. Psychol. 12:99. 10.1002/(SICI)1099-0720(199804)12:2<99::AID-ACP509>3.0.CO;2-7

[B137] Sagan C. (1979). Broca's Brain: The Romance of Science. New York, NY: Random House, 317–319.

[B138] Sagan C. Druyan A. (1997). The Demon-Haunted World: Science as a Candle in the Dark. New York, NY: Ballantine Books.

[B139] Schacter D. L. (1999). The seven sins of memory: Insights from psychology and cognitive neuroscience. Am. Psychol. 54, 182–203. 10.1037/0003-066X.54.3.182 10199218

[B140] Schacter D. L. (2001). The Seven Sins of Memory: How the Mind Forgets and Remembers. Houghton, Mifflin and Company.

[B141] Scherer K. R. Schorr A. Johnstone T. (2001). Appraisal Processes in Emotion: Theory, Methods, Research. Oxford: Oxford University Press.

[B142] Schmidt S. Muzzulini B. Levine L. J. Tinti C. (2021). Sources of bias in memory for emotional reactions to Brexit: current feelings mediate the link between appraisals and memories. Appl. Cogn. Psychol. 35, 819–827. 10.1002/acp.3806

[B143] Schmidt S. Tinti C. Levine L. J. Testa S. (2010). Appraisals, emotions and emotion regulation: an integrative approach. Motiv. Emot. 34, 63–72. 10.1007/s11031-010-9155-z 20376165 PMC2844958

[B144] Schnall S. Haidt J. Clore G. L. Jordan A. H. (2015). Landy and Goodwin (2015) confirmed most of our findings then drew the wrong conclusions. Perspect. Psychol. Sci. 10, 537–538. 10.1177/1745691615589078 26177952

[B145] Schoch S. F. Cordi M. J. Rasch B. (2017). Modulating influences of memory strength and sensitivity of the retrieval test on detectability of the sleep consolidation effect. Neurobiol. Learn. Mem. 145, 181–189. 10.1016/j.nlm.2017.10.009 29030296

[B146] Schrader G. Davis A. Stefanovic S. Christie P. (1990). The recollection of affect. Psychol. Med. 20, 105–109. 10.1017/S0033291700013271 2320688

[B147] Slabu L. Guinote A. (2010). Getting what you want: power increases the accessibility of active goals. J. Exp. Soc. Psychol. 46, 344–349. 10.1016/j.jesp.2009.10.013

[B148] Smith P. K. Bargh J. A. (2008). Nonconscious effects of power on basic approach and avoidance tendencies. Soc. Cogn. 26, 1–24. 10.1521/soco.2008.26.1.1 18568085 PMC2435045

[B149] Southwick S. M. Morgan C. A. Nicolaou A. L. Charney D. S. (1997). Consistency of memory for combat-related traumatic events in veterans of Operation Desert Storm. Am. J. Psychiatry 154, 173–177. 10.1176/ajp.154.2.173 9016264

[B150] Steblay N. (1992). A meta-analytic review of the weapon focus effect. Law Hum. Behav. 16, 413–424. 10.1007/BF02352267

[B151] Steele B. Martin M. Yakubovich A. Humphreys D. K. Nye E. (2022). Risk and protective factors for men's sexual violence against women at higher education institutions: a systematic and meta-analytic review of the longitudinal evidence. Trauma Violence Abuse 23, 716–732. 10.1177/1524838020970900 33176596 PMC9210109

[B152] Steele C. M. Josephs R. A. (1990). Alcohol myopia: its prized and dangerous effects. Am. Psychol. 45:921. 10.1037/0003-066X.45.8.921 2221564

[B153] Stephan W. Berscheid E. Walster E. (1971). Sexual arousal and heterosexual perception. J. Pers. Soc. Psychol. 20, 93–101. 10.1037/h0031943 5121168

[B154] Stevenson R. J. Case T. I. Oaten M. J. (2011). Effect of self-reported sexual arousal on responses to sex-related and non-sex-related disgust cues. Arch. Sex. Behav. 40, 79–85. 10.1007/s10508-009-9529-z 19705272

[B155] Strand R. W. Heitman L. D. (2017). The Forensic Experiential Trauma Interview (FETI). Arizona Coalition for Victim Services. Available online at: http://www.azcvs.net/wp-content/uploads/FETI-Public-Description-Jan-2017.pdf

[B156] Talarico J. M. Rubin D. C. (2003). Confidence, not consistency, characterizes flashbulb memories. Psychol. Sci. 14, 455–461. 10.1111/1467-9280.0245 12930476

[B157] Talarico J. M. Rubin D. C. (2007). Flashbulb memories are special after all; in phenomenology, not accuracy. Appl. Cogn. Psychol. 21, 557–578. 10.1002/acp.1293

[B158] Taylor A. Jordan K. Zajac R. Takarangi M. K. T. Garry M. (2020). Judgments of memory coherence depend on the conditions under which a memory is retrieved, regardless of reported PTSD symptoms. J. Appl. Res. Mem. Cogn. 9, 396–409. 10.1037/h0101855

[B159] Tippett K. (2021). Bessel van der Kolk: How trauma lodges in the body, revisited. On Being. Available online at: https://onbeing.org/programs/bessel-van-der-kolk-how-trauma-lodges-in-the-body-revisited/ (accessed June 26, 2024).

[B160] Trammell J. P. Clore G. L. (2014). Does stress enhance or impair memory consolidation? Cogn. Emot. 28, 361–374. 10.1080/02699931.2013.822346 23895111 PMC4096032

[B161] van der Kolk B. (2014). The Body Keeps the Score: Brain, Mind, and Body in the Healing of Trauma. London: Penguin Books.

[B162] van der Kolk B. A. (1998). The psychology and psychobiology of developmental trauma. Prax. Kinderpsychol. Kinderpsychiatr. 47, 19–35. 9522593

[B163] van der Kolk B. A. Fisler R. (1995). Dissociation and the fragmentary nature of traumatic memories: overview and exploratory study. J. Trauma. Stress 8, 505–525. 10.1002/jts.2490080402 8564271

[B164] van Leeuwen F. Dukes A. Tybur J. M. Park J. H. (2017). Disgust sensitivity relates to moral foundations independent of political ideology. Evol. Behav. Sci. 11:92. 10.1037/ebs0000075

[B165] Vanderploeg R. D. Donnell A. J. Belanger H. G. Curtiss G. (2014). Consolidation deficits in traumatic brain injury: the core and residual verbal memory defect. J. Clin. Exp. Neuropsychol. 36, 58–73. 10.1080/13803395.2013.864600 24303929

[B166] Villalobos J. G. Davis D. Leo R. A. (2016). History, Her Story: Sexual Miscommunication, Motivated Remembering, and Intoxication as Pathways to Honest FALSE Testimony Regarding Sexual Consent. Vilified: Wrongful Allegations of Sexual and Child Abuse. (Oxford: Oxford University Press, 2016, Forthcoming)., Univ. of San Francisco Law Research Paper, (2014-33). Available online at: https://papers.ssrn.com/sol3/papers.cfm?abstract_id=2480049 (accessed June 26, 2024).

[B167] Wagenaar W. A. Groeneweg J. (1990). The memory of concentration camp survivors. Appl. Cogn. Psychol. 4, 77–87. 10.1002/acp.2350040202

[B168] Wertheimer A. (2003). Consent to Sexual Relations. Cambridge: Cambridge University Press.

[B169] Whitson J. A. Liljenquist K. A. Galinsky A. D. Magee J. C. Gruenfeld D. H. Cadena B. (2013). The blind leading: power reduces awareness of constraints. J. Exp. Soc. Psychol. 49, 579–582. 10.1016/j.jesp.2012.10.009

[B170] Willis M. Blunt-Vinti H. D. Jozkowski K. N. (2019). Associations between internal and external sexual consent in a diverse national sample of women. Pers. Individ. Dif. 149, 37–45. 10.1016/j.paid.2019.05.029

[B171] Wilson L. C. Miller K. E. (2016). Meta-analysis of the prevalence of unacknowledged rape. Trauma Violence Abuse 17, 149–159. 10.1177/1524838015576391 25784571

[B172] Wood E. F. Rikkonen K. J. Davis D. (2019). “Definition, communication, and interpretation of sexual consent,” in Handbook of Sexual Assault and Sexual Assault Prevention, 1st Edn, eds. W. T. O'Donohue and P. A. Schewe (New York, NY: Springer), 399–421.

[B173] Yu A. N. C. Iodice P. Pezzulo G. Barca L. (2021). Bodily information and top-down affective priming jointly affect the processing of fearful faces. Front. Psychol. 12:625986. 10.3389/fpsyg.2021.625986 34149514 PMC8206275

[B174] Zurbriggen E. L. (2000). Social motives and cognitive power-sex associations: predictors of aggressive sexual behavior. J. Pers. Soc. Psychol. 78, 559–581. 10.1037/0022-3514.78.3.559 10743881

